# A Tribute to Evgenii V. Ananiev, 1947–2008

**DOI:** 10.1371/journal.pgen.1000122

**Published:** 2008-08-29

**Authors:** Sergei M. Mirkin

**Affiliations:** Tufts University, Medford, Massachusetts, United States of America; Stanford University, United States of America

**Image 1:**
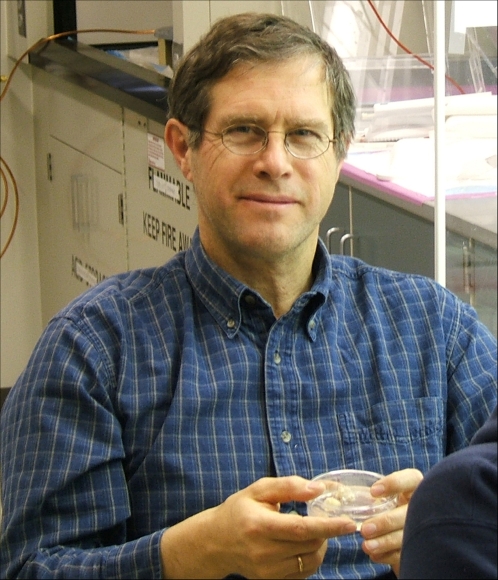
Evgenii Ananiev.

This article is a tribute to Dr. Evgenii Ananiev, who passed away on January 10, 2008, after a year-long battle with a brain tumor. It is nearly impossible for me to comprehend this loss, as Evgenii (Image 1) was always larger than life—full of ideas, vitality, and joy. I first met Evgenii more than 30 years ago, when I started working on my senior thesis in the laboratory of Roman Khesin in the legendary Radiobiology Department of the Atomic Energy Institute in Moscow. Evgenii was a postdoctoral fellow in the lab next door headed by Vladimir Gvozdev. From day one, it was hard not to notice this energetic, well-built young man with a broad, charismatic smile and engaging manners. He would bump into you in the corridor to share his excitement about his most recent observations made using DNA hybridization in situ with the polytene chromosomes of *Drosophila*. Admittedly, he had every reason to get excited, as his data provided the first molecular evidence for the existence of mobile elements in eukaryotes—a discovery of foremost significance. The paper describing these striking results came out in *Science*, an unprecedented success for Soviet molecular biology.

Scientific authority aside, Evgenii was married to an extremely talented and equally beautiful young scientist, Olga Danilevskaya, whom every other man in the Institute secretly admired. Evgenii and Olga liked to throw parties for the two labs in their modest apartment in the outskirts of Moscow: red wine brought by Olga from her native Crimea, a bucket of Russian salad, passionate discussions of science and politics until early dawn, some guitar singing, Evgenii filming high points of the evening—moments of happiness…

Evgenii's path to science was far from ordinary. He was born in Moscow in 1947 into a working-class family. His parents were typical Russian children of World War II, who didn't have a chance to complete their high school education because they started working at the military factories in their teens. Science was not high on the family priority list, and there were no indications that Evgenii would go there. At the age of 10, however, Evgenii had an unusual revelation: he realized that he was only alive as long as his heart continued beating. He decided at that very moment that he would become a biologist to deal with the mortality problem once and for all. This naïve, childhood impulse eventually transformed into a genuine interest in biology, which became a cornerstone of Evgenii's life. It didn't come easily, however. Upon his parents' divorce, Evgenii had to go to work to help his mother financially. He managed to continue his education, first in night high school, and later in the night program at Moscow State University, but he dearly needed more guidance.

A golden opportunity came when Evgenii was hired as a technician in the laboratory of Alexandra Prokofyeva-Belgovskaya at the Institute of Molecular Biology, Union of Soviet Socialist Republics Academy of Sciences. To fully appreciate his luck, one should understand the status of Prokofyeva-Belgovskaya in Russian science. She was a world-renowned cytogeneticist who, in collaboration with Hermann Muller, made principal contributions toward the understanding of chromosome structure in the 1930s. Her lab was by far the best place to study cytogenetics in Russia. Evgenii's first mentor was an outstanding young scientist, Victor Gindilis, who remained his friend for the rest of his life. It was in this lab that Evgenii learned the fine art of cytology and microscopy and developed his life-long interest in chromosome biology.

Evgenii's hard work and the help from the lab members bore fruit: he transferred to the day-time Biology program at the Moscow State University and graduated in 1970 with a B.S. in Genetics. For his Ph.D. studies, he joined the laboratory of Vladimir Gvozdev at the Institute of Atomic Energy in Moscow. This was quite a switch from his previous environment, as Vladimir was in effect a starting Assistant Professor back then. His lab was full of talented and devoted young scientists, who worked on all kinds of exciting problems. This was a terrific environment for Evgenii, who started working on three big projects at once: changes in transcription and replication associated with the position variegation effect in *Drosophila*, dosage compensation in *Drosophila* X-chromosomes, and chromosomal replication in fly cell culture. In just three years, he published five experimental papers and defended his Ph.D. thesis in 1975. He then decided to stay for his postdoctoral studies in the same lab. The stars were aligning for his major discovery.

**Figure 1:**
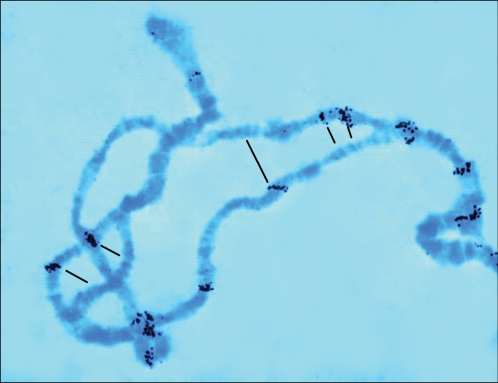
Differential hybridization of a mobile genetic element with asynaptic regions of *Drosophila* polytene Chromosome 3.

Around this time, Georgii Georgiev initiated studies of moderately repeated elements of the *Drosophila* genome in his lab at the Institute of Molecular Biology, USSR Academy of Sciences and set up the collaboration with Gvozdev's lab. Nikolai Tchurikov and Yurii Ilyin had cloned and characterized several such elements, while Evgenii was mapping their location in *Drosophila* polytene chromosomes by in situ hybridization. Evgenii later described his principal discovery as such:

“On my very first slide, I observed hybridization of a particular DNA clone with roughly 40 sites in *Drosophila* polytene chromosomes…. One nucleus on the slide attracted my particular attention. In this nucleus, paternal and maternal chromosomes were not conjugated along their whole lengths, as is typical for polytene chromosomes, but were separated locally. Unexpectedly, the distribution of hybridization sites on these separated homologues was totally different (Figure 1)!… I thought that this phenomenon might be due to the fact that I deliberately used exceptionally large larvae obtained by crossing two different *Drosophila* strains. Thus, the asymmetric distribution of the hybridization sites could simply reflect the differences in the element's location between the parental chromosomes….I then analyzed every case of homologous chromosome asynapsis and found that uneven distribution of hybridization sites between the parental chromosomes was always the case….It soon occurred to me that our DNA elements could, in fact, be ‘jumping genes’!”

A paper from the two labs describing these unusual DNA elements came out in *Science* in 1977 [1]. It integrated Evgenii's idea that “The unstable localization of the repeated sequences in polytenic chromosomes may be related to the phenomenon of gene migration….” Two years later, these observations were confirmed and expanded by Gerald Rubin and his colleagues at Harvard Medical School, who called these *Drosophila* elements mobile genes [2],[3]. Evgenii's pioneering studies of mobile genes in flies were well acknowledged in his home country: he was habilitated as Doctor of Sciences in 1983 and granted the highest Russian scientific prize—the National Award for Science.

Soon thereafter, Evgenii's career took a new path. He was invited to head a laboratory of plant molecular genetics at the Institute of General Genetics, USSR Academy of Sciences, which he saw as a unique opportunity to establish his independent research program. Here, Evgenii was exposed to plant molecular genetics for the first time. Despite scarce resources and a worsening economic situation in the country, he initiated intensive studies of barley plant genomics, one of which led to the characterization of a barley-specific repeated DNA element, called *Dialect*. The demise of Russian science following the disintegration of the Soviet Union put this promising research on hold, however. And in 1992, Evgenii immigrated to the US hoping to continue his scientific career.

After a temporary stay at Brandeis University, Evgenii became fascinated by the so-called oat–maize chromosome addition lines, which were developed in Ron Phillips and Howard Rines' labs, and joined their project at the University of Minnesota in 1995. This was a fantastic choice: the potential of these chromosome addition lines for the physical mapping of maize DNA was unmatched; Ron and Howard immediately appreciated Evgenii's scientific might and ingenuity; and everyone in the lab reveled in the opportunity for a breakthrough in maize genetics.

The next three years were probably the most productive period in Evgenii's career. To isolate genetic elements from individual maize chromosomes, he used his old mantra—mobile elements. From his work in *Drosophila*, he knew that retrotransposons are highly species-specific. He decided, therefore, to screen DNA libraries obtained from the oat–maize chromosome addition lines with a probe containing a mixture of common maize retrotransposons. The strategy worked beautifully, resulting in collections of chromosome-specific maize genes.

His next big goal was to characterize centromeres of maize chromosomes. Graham Moore from the John Innes Centre had just described a wheat centromeric sequence that appeared to hybridize with centromeres of rice and maize as well. Using this sequence as a probe, Evgenii isolated DNA fragments corresponding to the centromere of maize Chromosome 9. Subsequent analysis led him to conclude that maize centromeres are built from multiple tandem copies of a minisatellite, which he called *CentC,* that are occasionally interrupted by retrotransposons. Evgenii's paper on the molecular organization of maize centromeres [4] was a seminal contribution to the field.

In 1998, Evgenii became a Research Scientist at the Pioneer Hi-Bred Company, where he and his group were charged with building the physical map of the maize genome. His ambition, however, was to create a maize artificial chromosome from the individual building blocks, i.e., replication origins, centromeres, telomeres, and appropriate selective markers. By 2006, his group had succeeded in creating the first plant artificial chromosome. It looked like a true novel chromosome: small, but otherwise morphologically indistinguishable from the rest of the maize chromosomes. Unfortunately, a horrible disease stopped Evgenii at the moment he considered the highest point of his career.

It is symbolic that Evgenii's scientific path started in 1964 as a lab technician doing morphometric analysis of chromosomes and ended with the construction of the artificial chromosome some 40 years later. He published 70 papers during his career, many of which shaped the field of chromosome biology. His passion for science was an inspiration for his many friends, pupils, and colleagues. Yet Evgenii was an exceptionally gifted person in many other regards. He had a flair for filmmaking, photography, painting, and slideshows. Defying disease, he continued his scientific and artistic pursuits until his very last days. Paraphrasing a popular writer of Evgenii's youth, the man was not made for defeat; he could be destroyed but not defeated.
